# Supplementary material for: Splicing Modulation Results in Aberrant Isoforms and Protein Products of p53 Pathway Genes and the Sensitization of B Cells to Non-Genotoxic MDM2 Inhibition
Source: Int J Mol Sci. 2023 Jan 26;24(3):2410. doi: 10.3390/ijms24032410 (PMC9916657; doi:10.3390/ijms24032410)
Supplement: Supplementary file 1 [file ijms-24-02410-s001.zip › ijms-2135245-supplementary.pdf]

Supplementary Table S1. Cell lines

Cell Line	Cell Type	Origin	[1]*TP53* status	[2]IC_{50} [nM, E7107]
**Ramos**	B lymphocyte	Burkitt's lymphoma	**Mutant**<br>(Homozygous)<br>c.761T>A; p.I254N	14.2 ± 2.6
**Raji**	B lymphocyte	Burkitt's lymphoma	**Mutant**<br>(Heterozygous)<br>c.638G>A; p.R213Q<br>c.700T>C; p.Y234H	4.1 ± 1.2
**Pfeiffer**	B lymphocyte	B-cell non-Hodgkin's lymphoma	**Null**<br>c.(del)	5.1 ± 0.5
**Daudi**	B lymphocyte	Burkitt's lymphoma	**Mutant**<br>(Heterozygous)<br>c.797G>A; p.G266E<br>c.637C>T; p.R213W	6.3 ± 0.5
**Nalm-6**	B cell precursor leukemia	Acute lymphoblastic leukemia (ALL)	**Wild type**	1.8 ± 0.1
**OCI-Ly3**	B lymphocyte	B-cell non-Hodgkin’s lymphoma	**Wild type**	4.2 ± 1.2
**HAL-01**	B cell precursor leukemia	Acute lymphoblastic leukemia (ALL)	**Wild type**	203.5 ± 14.3
**HEL**	Erythroblast	Erythroleukemia	**Mutant**<br>(Homozygous)<br>c.398T>A; p.M133K	60.2 ± 2.9

[1]The genetic status of *TP53* in these cell lines was obtained from IARC TP53 database and COSMIC (Catalogue of Somatic Mutations in Cancer) database.
[2]This shows the mean of n=3 independent repeats ± SEM.

Supplementary Table S2. IC_{50} values of E7107 for primary CLL samples and normal PBMCs

Clinical information				p53 status		*SF3B1*	
**Tumour ID**	**Age**	**Sex**	**Cytogenetic abnormalities**	***TP53* status (NGS)**	**LC_{50} RG7388 (μM)**	***SF3B1* status (Sanger)**	**LC_{50} E7107 (nM)**
**275**	88	M	none	WT	0.88	WT	**20.3**
**276**	67	M	none	WT	1.7	WT	**6.2**
**281**	68	M	del13q	c.623A>T, p.D208V; c.659A>G, p.Y220C	8.8	WT	**8.6**
**282**	81	F	del13q	WT	0.63	WT	**5.9**
**283**	69	F	none	c.745A>G, p.R249G	9.2	c.2225G>A, p.G742D	**9.9**
**284**	63	F	none	WT	0.56	WT	**17.8**
**287**	69	M	del13q,del11q	c.524G>A, p.R175H	2.7	c.2098A>G, p.K700E	**6.3**
**288**	72	F	none	WT	>10	c.2219G>A, p.G740E	**6.6**
**289**	77	M	none	WT	0.65	WT	**8.8**
**291**	84	M	none		0.88	WT	**26**
**292**	66	M	none		0.51	WT	**3.3**
**293**	57	M	del13q		0.32	WT	**6.7**
**296**	69	M	none		2.3	WT	**7.4**
**297**	83	F	none		9.3	WT	**6.8**
**298**	77	M	none		2.3	WT	**6.2**
**301**	54	M	none		0.22	WT	**5.5**
**302**	73	M	none		0.56	WT	**5.6**
**305**	81	M	none		0.31	c.2223G>C, p.K741N	**7.4**
**306**	77	F	none		0.75	WT	**2.9**
**308**	74	M	none		0.4	WT	
**PBMC1**		M			>10		**>300**
**PBMC2**		M			>10		**>300**
**PBMC3**		F			>10		**>300**
**WT:** wild type. Blank boxes indicate that there is no data available.							

Supplementary Figure S1. Sequencing chromatogram of *MDM2* transcript showing multi-exon skipping of exons 3-10

cDNA sequencing of MDM2 showing multi-exon skipping of exons 3-10

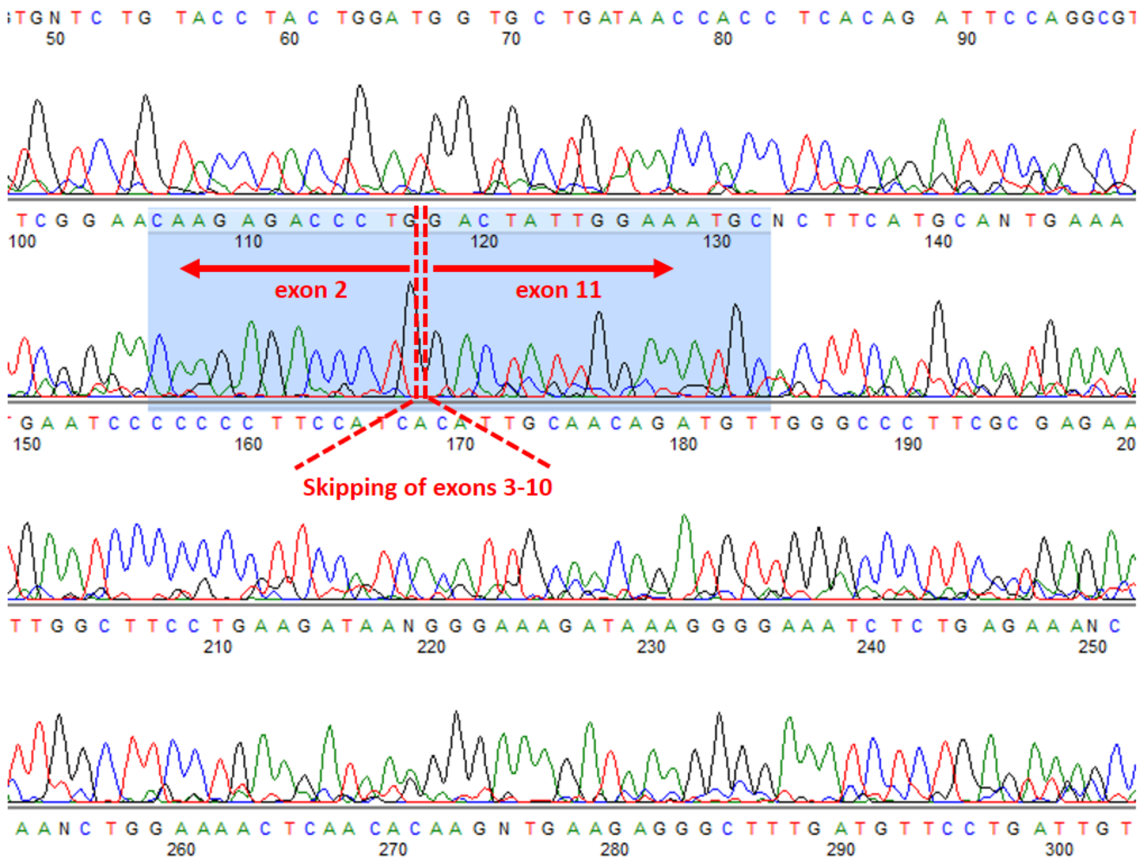

Supplementary Figure S2. Sequence and alignment evidence of *CDKN1A* transcript (P21^{L})

Supplementary Figure S2A. Homo sapiens *cyclin dependent kinase inhibitor 1A* (*CDKN1A*), RefSeqGene on chromosome 6

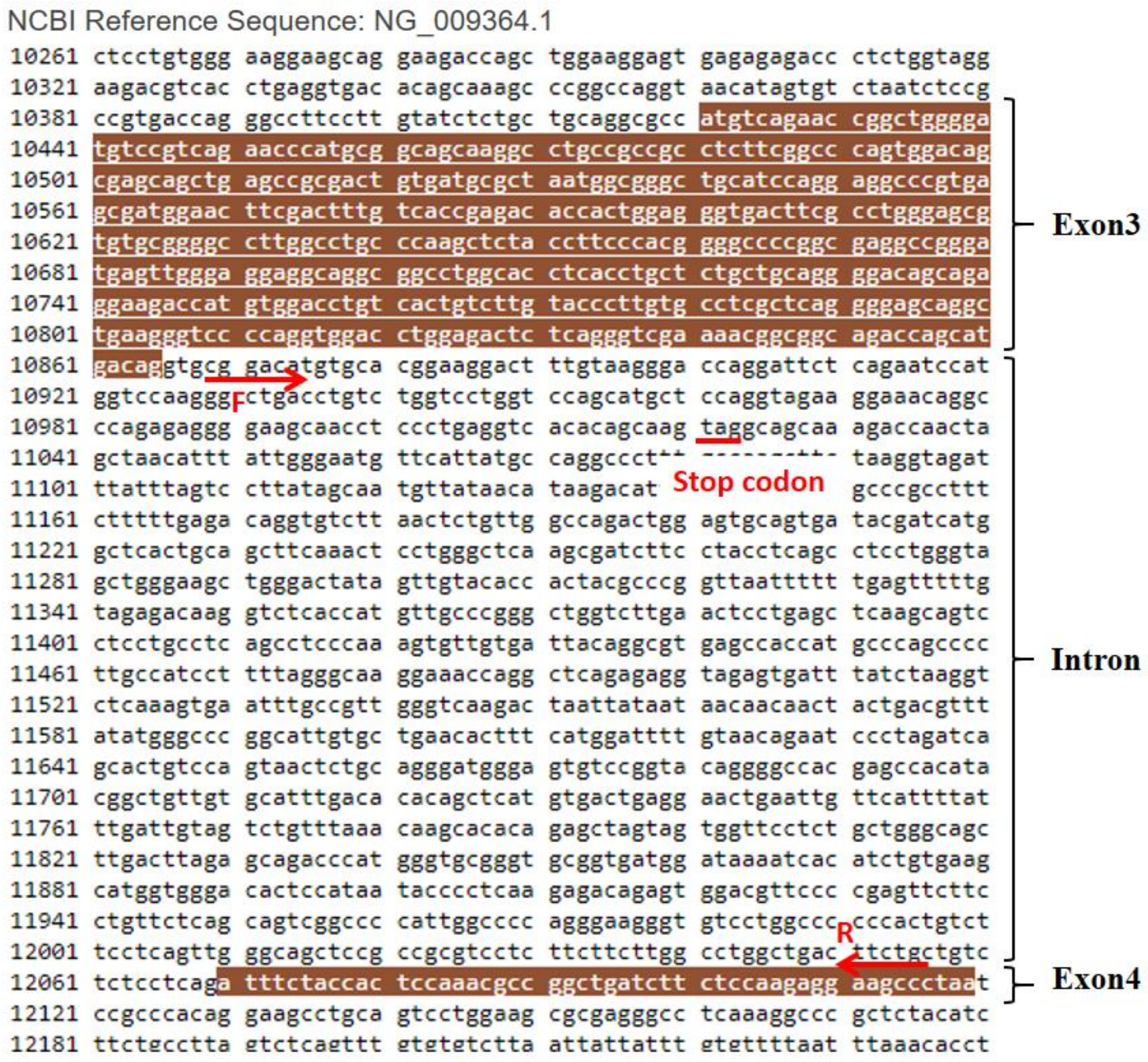

Supplementary Figure S2B. Sequence alignment of *CDKN1A* transcript (P21^{L}) spanning exons 3 to 4 (Forward)

Homo sapiens chromosome 6, GRCh38.p12 Primary Assembly

Sequence ID: NC_000006.12 **Length:** 170805979 **Number of Matches:** 1

Range 1: 36684550 to 36685269 GenBank Graphics ▼ Next Match ▲ Previous Match

Score	Expect	Identities	Gaps	Strand
1162 bits(629)	0.0	699/738(95%)	19/738(2%)	Plus/Plus

Features: cyclin-dependent kinase inhibitor 1 isoform 2
cyclin-dependent kinase inhibitor 1 isoform 1

```
Query  12        CGGA-ATGAGGCACGGNAAGGANTTTGNTAAGGGACCAGGATTCTCAGTAATCCATGGCN  70
                 |||| |||  |||||| ||||| |||| |||||||||||||||||||| |||||||||
Sbjct  36684550  CGGACATG-TGCACGG-AAGGACTTTG-TAAGGGACCAGGATTCTCAG-AATCCATGG-T  36684604

Query  71        CCANGGGCTGACCTGNTCTGGTCCTGGTCCAGCATGCTCCAGGCTAGAaggaaacaggcc  130
                 ||| ||||||||||| ||||||||||||||||||||||||||| ||||||||||||||||
Sbjct  36684605  CCAAGGGCTGACCTG-TCTGGTCCTGGTCCAGCATGCTCCAGG-TAGAAGGAAACAGGCC  36684662

Query  131       cagagaggggaagcaacctccctgaggtcacacagcaagtaggcagcaaagACCAACTAG  190
                 ||||||||||||||||||||||||||||||||||||||||||||||||||||||||||||
Sbjct  36684663  CAGAGAGGGGAAGCAACCTCCCTGAGGTCACACAGCAAGTAGGCAGCAAAGACCAACTAG  36684722

Query  191       CTAACATTTATTGGGAATGTTCATTATGCCAGGCCCTTTGCCAAGCTTCTAAGGTAGATT  250
                 ||||||||||||||||||||||||||||||||||||||||||||||||||||||||||||
Sbjct  36684723  CTAACATTTATTGGGAATGTTCATTATGCCAGGCCCTTTGCCAAGCTTCTAAGGTAGATT  36684782

Query  251       TATTTAGTCCTTATAGCAATGTTATAACATAAGACATTCTTGTCACCCTGCCCGCCtttc  310
                 ||||||||||||||||||||||||||||||||||||||||||||||||||||||||||||
Sbjct  36684783  TATTTAGTCCTTATAGCAATGTTATAACATAAGACATTCTTGTCACCCTGCCCGCCTTTC  36684842

Query  311       tttttgagacaggtgtcttaactctgttggccagactggagtgcagtgatacgatcatgg  370
                 ||||||||||||||||||||||||||||||||||||||||||||||||||||||||||||
Sbjct  36684843  TTTTTGAGACAGGTGTCTTAACTCTGTTGGCCAGACTGGAGTGCAGTGATACGATCATGG  36684902

Query  371       ctcactgcagcttcaaactcctgggctcaagcgatcttcctacctcagcctcctgggtag  430
                 ||||||||||||||||||||||||||||||||||||||||||||||||||||||||||||
Sbjct  36684903  CTCACTGCAGCTTCAAACTCCTGGGCTCAAGCGATCTTCCTACCTCAGCCTCCTGGGTAG  36684962

Query  431       ctgggaagctgggactatagttgtacaccactacgcccggttaatttttgagtttttgt  490
                 ||||||||||||||||||||||||||||||||||||||||||||||||||||||||||||
Sbjct  36684963  CTGGGAAGCTGGGACTATAGTTGTACACCACTACGCCCGGTTAATTTTTGAGTTTTTGT  36685022

Query  491       anagacaaggtctcaccatgttgcccggnctggtcttgaactcctgagctcaagcagtcc  550
                 | |||||||||||||||||||||||||| |||||||||||||||||||||||||||||||
Sbjct  36685023  AGAGACAAGGTCTCACCATGTTGCCCGGGCTGGTCTTGAACTCCTGAGCTCAAGCAGTCC  36685082

Query  551       tcctgcctcancctcccaaagtgtngtgattacaggcgtgagccaccatgcccagccCCT  610
                 |||||||||| ||||||||||||| |||||||||||||||||||||||||||||||||||
Sbjct  36685083  TCCTGCCTCAGCCTCCCAAAGTGTTGTGATTACAGGCGTGAGCCACCATGCCCAGCCCCT  36685142

Query  611       TGCCATCCTTTTAGGGCAAGGAAAACCAGGCTCAGAGAGGTAGNAGTGATTNATCTAAGG  670
                 ||||||||||||||||||||| ||||||||||||||||||||| ||||||| ||||||||
Sbjct  36685143  TGCCATCCTTTTAGGGCAAGG-AAACCAGGCTCAGAGAGGTAG-AGTGATTTATCTAAGG  36685200

Query  671       NCTNCAANGGNAATTNGTCCGTNGGGNCCAAGACTNATTNNTAATAANCAACNACCTACT  730
                  || ||| |  |||| | |||| ||| | |||||| |||  |||||| |||| || ||||
Sbjct  36685201  TCT-CAAAGTGAATTTG-CCGTTGGGTC-AAGACTAATTA-TAATAA-CAACAAC-TACT  36685254

Query  731       GGACNTTTANTATTGGGC  748
                 | || |||| ||| ||||
Sbjct  36685255  G-ACGTTTA-TAT-GGGC  36685269
```

Supplementary Figure S2C. Sequence alignment of *CDKN1A* transcript (P21[L]) spanning exons 3 to 4 (Reverse)

Homo sapiens chromosome 6, GRCh38.p12 Primary Assembly

Sequence ID: NC_000006.12 **Length:** 170805979 **Number of Matches:** 1

Range 1: 36685008 to 36685736 GenBank Graphics ▼ Next Match ▲ Previous Match

Score	Expect	Identities	Gaps	Strand
1147 bits(621)	0.0	688/737(93%)	8/737(1%)	Plus/Minus

Features: cyclin-dependent kinase inhibitor 1 isoform 2
cyclin-dependent kinase inhibitor 1 isoform 1

```
Query  22        CAGAAGTCCCCCAGGCCAAGCAAGAAGAGGACGCGGCGGNAGCTGCCCAACTGTANGAAG  81
                 ||||||||  |||||||||| |||||||||||||||||| ||||||||||||| | ||||
Sbjct  36685736  CAGAAGTCAGCCAGGCCAAG-AAGAAGAGGACGCGGCGG-AGCTGCCCAACTG-AGGAAG  36685680

Query  82        ACAGTTGGGGGGCCAGGACACCCTTCCCTGGGGCCAATGGGGCCGACTGCTGAGAACAGG  141
                 |||| |||||||||||||||||||||||||||||||||||||||||||||||||||||||
Sbjct  36685679  ACAG-TGGGGGGCCAGGACACCCTTCCCTGGGGCCAATGGGGCCGACTGCTGAGAACAGG  36685621

Query  142       AAGAACTCGGGGAACGTCCACTCTGATCTCTTGAGGGGTATTATGGAGTGTCCCACCATG  201
                 ||||||||||||||||||||||||| ||||||||||||||||||||||||||||||||||
Sbjct  36685620  AAGAACTCGGGGAACGTCCACTCTG-TCTCTTGAGGGGTATTATGGAGTGTCCCACCATG  36685562

Query  202       CTTCACAGATGTGATTTTATCCATCACCGCACCCGCACCCATGGGTCTGCTCTAAGTCAA  261
                 ||||||||||||||||||||||||||||||||||||||||||||||||||||||||||||
Sbjct  36685561  CTTCACAGATGTGATTTTATCCATCACCGCACCCGCACCCATGGGTCTGCTCTAAGTCAA  36685502

Query  262       GCTGCCCAGCAGAGGAACCACTACTAGCTCTGTGTGCTTGTTTAAACAGACTACANTCAN  321
                 ||||||||||||||||||||||||||||||||||||||||||||||||||||||| ||| 
Sbjct  36685501  GCTGCCCAGCAGAGGAACCACTACTAGCTCTGTGTGCTTGTTTAAACAGACTACAATCAA  36685442

Query  322       NTAAANTGAACAATTCNNTTCCTCAGTCACATGANCTGTGTGTCAAATGCACAACAGNCG  381
                  |||| ||||||||||  |||||||||||||||| |||||||||||||||||||||| ||
Sbjct  36685441  ATAAAATGAACAATTCAGTTCCTCAGTCACATGAGCTGTGTGTCAAATGCACAACAGCCG  36685382

Query  382       TATGTGGCTCGTGGCCCCTGTACCNGACACTCCCATCCCTGCANANTTACTGGACANTGC  441
                 |||||||||||||||||||||||| |||||||||||||||||| | |||||||||| |||
Sbjct  36685381  TATGTGGCTCGTGGCCCCTGTACCGGACACTCCCATCCCTGCAGAGTTACTGGACAGTGC  36685322

Query  442       TGATCTAGGGATTCTGTTACAAAATCCATGAAAGTGTTCAGCACAATGCCGGGCCCATAT  501
                 ||||||||||||||||||||||||||||||||||||||||||||||||||||||||||||
Sbjct  36685321  TGATCTAGGGATTCTGTTACAAAATCCATGAAAGTGTTCAGCACAATGCCGGGCCCATAT  36685262

Query  502       AAACGTCAGTAGTTGTTGTTATTATAATTAGTCTTGACCCAACGGCAAATTCActttgag  561
                 ||||||||||||||||||||||||||||||||||||||||||||||||||||||||||||
Sbjct  36685261  AAACGTCAGTAGTTGTTGTTATTATAATTAGTCTTGACCCAACGGCAAATTCACTTTGAG  36685202

Query  562       accttnagataaatcactctacctctctgagcctgggtttcctTGCCCTAAAANGATGGN  621
                 ||||| |||||||||||||||||||||||||||||| |||||||||||||||| ||||| 
Sbjct  36685201  ACCTT-AGATAAATCACTCTACCTCTCTGAGCCTGG-TTTCCTTGCCCTAAAAGGATGGC  36685144

Query  622       AAGGggctnggcatgatggctcacgcctgtaatcacnanacttngnnaggntgangcngn  681
                 |||||||| |||||| |||||||||||||||||||| | |||| |  ||| ||| || | 
Sbjct  36685143  AAGGGGCTGGGCATGGTGGCTCACGCCTGTAATCACAACACTTTGGGAGGCTGAGGCAGG  36685084

Query  682       agnantgcttgagctcangagttcanaaccngcccggntcaacatggtganaccttntct  741
                 || | ||||||||||| |||||||  ||| ||||||  ||||||||||| ||||| |||
Sbjct  36685083  AGGACTGCTTGAGCTCAGGAGTTCAAGACCAGCCCGGG-CAACATGGTGAGACCTTGTCT  36685025

Query  742       ttaaaaaaantnaaaaa  758
                  || ||||| | |||||
Sbjct  36685024  CTACAAAAACTCAAAAA  36685008
```

Supplementary Figure S3. Sequence evidence of the integrated provirus

Supplementary Figure S3A. Gel image result before sequencing

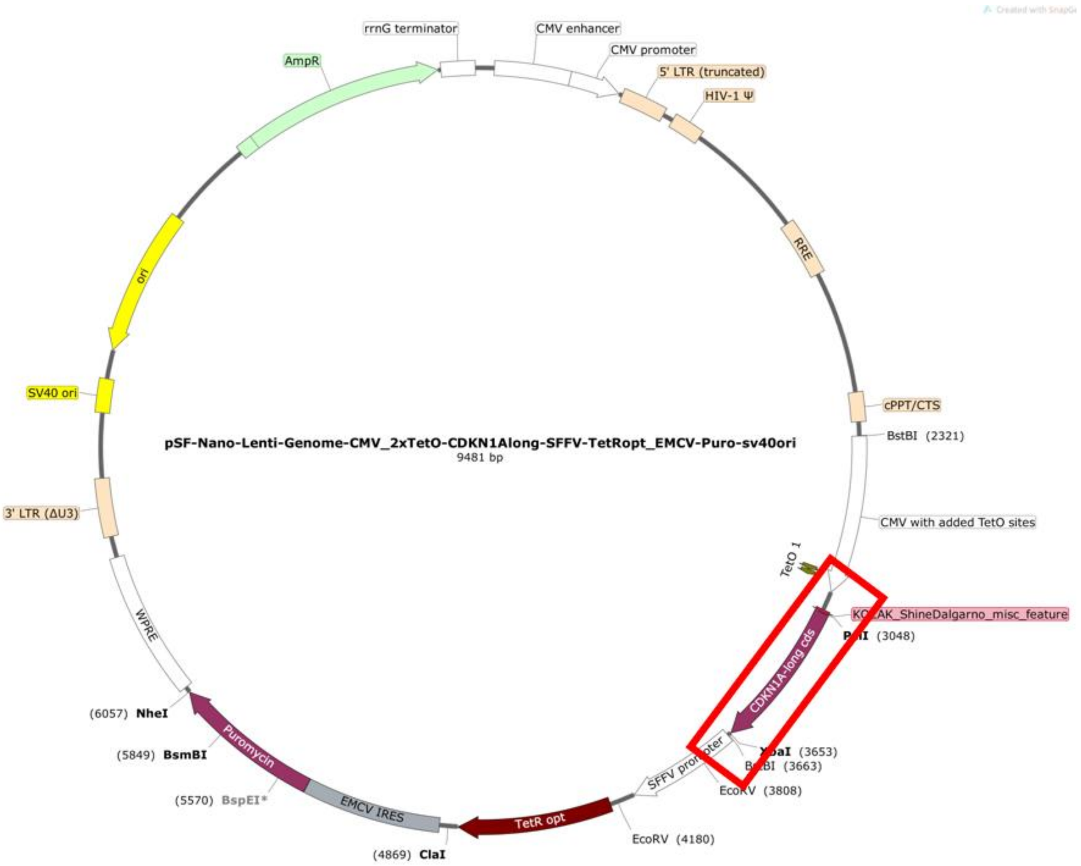

Primers to sequence integrated proviral DNA

F: 5` GCGTGTACGGTGGGAGGTC 3` (2850..2868)

R: 5` CTATCTTGCCAAACCTACAG 3` (3681..3700)

Product size: 850

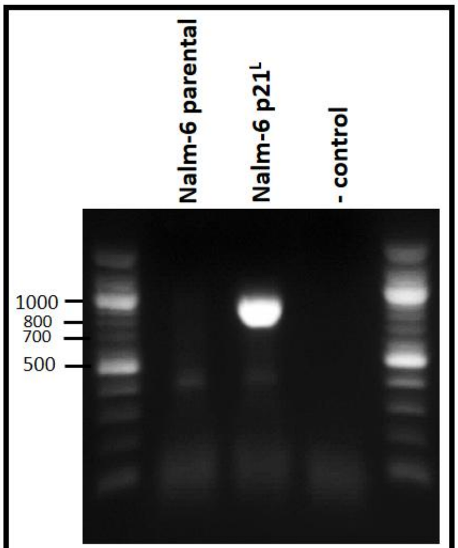

1.5% gel, 100V, 75min

Supplementary Figure S3B. Forward sequence alignment with reference sequence

Silent mutation created during cloning is shown in red box. A silent mutation of aspartic acid at position 151 was introduced in the longer CDKN1A sequence, changing it from GAC to GAT (see below). It was done in order to create a suitable sequence for restriction endonuclease cutting during cloning steps.

Download GenBank Graphics

Homo sapiens cyclin dependent kinase inhibitor 1A (CDKN1A), RefSeqGene on chromosome 6 **Forward**

Sequence ID: NG_009364.1 Length: 15651 Number of Matches: 1

Range 1: 10420 to 11022 GenBank Graphics ▼ Next Match ▲ Previous Match

Score	Expect	Identities	Gaps	Strand
1062 bits(575)	0.0	593/604(98%)	2/604(0%)	Plus/Plus

```
Query  187    CATGTCAGAACCGGCTGGGGATGTCCGTCAGAACCCATGCGGCAGCAAGGCCTGCCGCCG  246
              ||||||||||||||||||||||||||||||||||||||||||||||||||||||||||||
Sbjct  10420  CATGTCAGAACCGGCTGGGGATGTCCGTCAGAACCCATGCGGCAGCAAGGCCTGCCGCCG  10479

Query  247    CCTCTTCGGCCCAGTGGACAGCGAGCAGCTGAGCCGCGACTGTGATGCGCTAATGGCGGG  306
              ||||||||||||||||||||||||||||||||||||||||||||||||||||||||||||
Sbjct  10480  CCTCTTCGGCCCAGTGGACAGCGAGCAGCTGAGCCGCGACTGTGATGCGCTAATGGCGGG  10539

Query  307    CTGCATCCAGGAGGCCCGTGAGCGATGGAACTTCGACTTTGTCACCGAGACACCACTGGA  366
              ||||||||||||||||||||||||||||||||||||||||||||||||||||||||||||
Sbjct  10540  CTGCATCCAGGAGGCCCGTGAGCGATGGAACTTCGACTTTGTCACCGAGACACCACTGGA  10599

Query  367    GGGTGACTTCGCCTGGGAGCGTGTGCGGGGCCTTGGCCTGCCCAAGCTCTACCTTCCCAC  426
              ||||||||||||||||||||||||||||||||||||||||||||||||||||||||||||
Sbjct  10600  GGGTGACTTCGCCTGGGAGCGTGTGCGGGGCCTTGGCCTGCCCAAGCTCTACCTTCCCAC  10659

Query  427    GGGGCCCCGGCGAGGCCGGGATGAGTTGGGAGGAGGCAGGCGGCCTGGCACCTCACCTGC  486
              ||||||||||||||||||||||||||||||||||||||||||||||||||||||||||||
Sbjct  10660  GGGGCCCCGGCGAGGCCGGGATGAGTTGGGAGGAGGCAGGCGGCCTGGCACCTCACCTGC  10719

Query  487    TCTGCTGCAGGGGACAGCAGAGGAAGACCATGTGGACCTGTCACTGTCTTGTACCCTTGT  546
              ||||||||||||||||||||||||||||||||||||||||||||||||||||||||||||
Sbjct  10720  TCTGCTGCAGGGGACAGCAGAGGAAGACCATGTGGACCTGTCACTGTCTTGTACCCTTGT  10779

Query  547    GCCTCGCTCAGGGGAGCAGGCTGAAGGGTCCCCAGGTGGACCTGGAGACTCTCAGGGTCG  606
              ||||||||||||||||||||||||||||||||||||||||||||||||||||||||||||
Sbjct  10780  GCCTCGCTCAGGGGAGCAGGCTGAAGGGTCCCCAGGTGGACCTGGAGACTCTCAGGGTCG  10839

Query  607    AAAACGGCGGCAGACCAGCATGACAGGTGCGGATATGTGCACGGAAGGACTTTGTAAGGG  666
              ||||||||||||||||||||||||||||||||| ||||||||||||||||||||||||||
Sbjct  10840  AAAACGGCGGCAGACCAGCATGACAGGTGCGGACATGTGCACGGAAGGACTTTGTAAGGG  10899

Query  667    ACCAGGATTCTCAGAATCCATGGTCCAAGGGCTGACCTGTCTGGTCCTGGTCCAGCATGC  726
              ||||||||||||||||||||||||||||||||||||||||||||||||||||||||||||
Sbjct  10900  ACCAGGATTCTCAGAATCCATGGTCCAAGGGCTGACCTGTCTGGTCCTGGTCCAGCATGC  10959

Query  727    TCNAGGTAAAAGGAAACAGGCCANAANAGGGNAAGCAAC-TCCCNTGANGTCACACAGCA  785
              || ||||| |||||||||||||   | |||| ||||||| |||| ||| |||||||||||
Sbjct  10960  TCCAGGTAGAAGGAAACAGGCCCAGAGAGGGGAAGCAACCTCCC-TGAGGTCACACAGCA  11018

Query  786    AGTA  789
              ||||
Sbjct  11019  AGTA  11022
```

Supplementary Table S3. Concentrations of inhibitors used for combination treatment

		**0.25 x IC_{50}**	**0.5 x IC_{50}**	**1 x IC_{50}**	**2 x IC_{50}**	**4 x IC_{50}**
Nalm-6	RG7388 (µM)	0.0175	0.035	0.07	0.14	0.28
	E7107 (nM)	0.45	0.9	1.8	3.6	7.2
OCI-Ly3	RG7388 (µM)	0.0225	0.045	0.09	0.18	0.36
	E7107 (nM)	1.05	2.1	4.2	8.4	16.8
Nalm-6 $SF3B1^{K700K}$	RG7388 (µM)	0.035	0.07	0.14	0.28	0.56
	E7107 (nM)	0.75	1.5	3	6	12
Nalm-6 $SF3B1^{K700E}$	RG7388 (µM)	0.125	0.25	0.5	1	2
	E7107 (nM)	0.75	1.5	3	6	12

Supplementary Figure S4. Individual CLL samples (n=4) analysed for both single-drug and combination drug treatments using matrix assay

CLL294

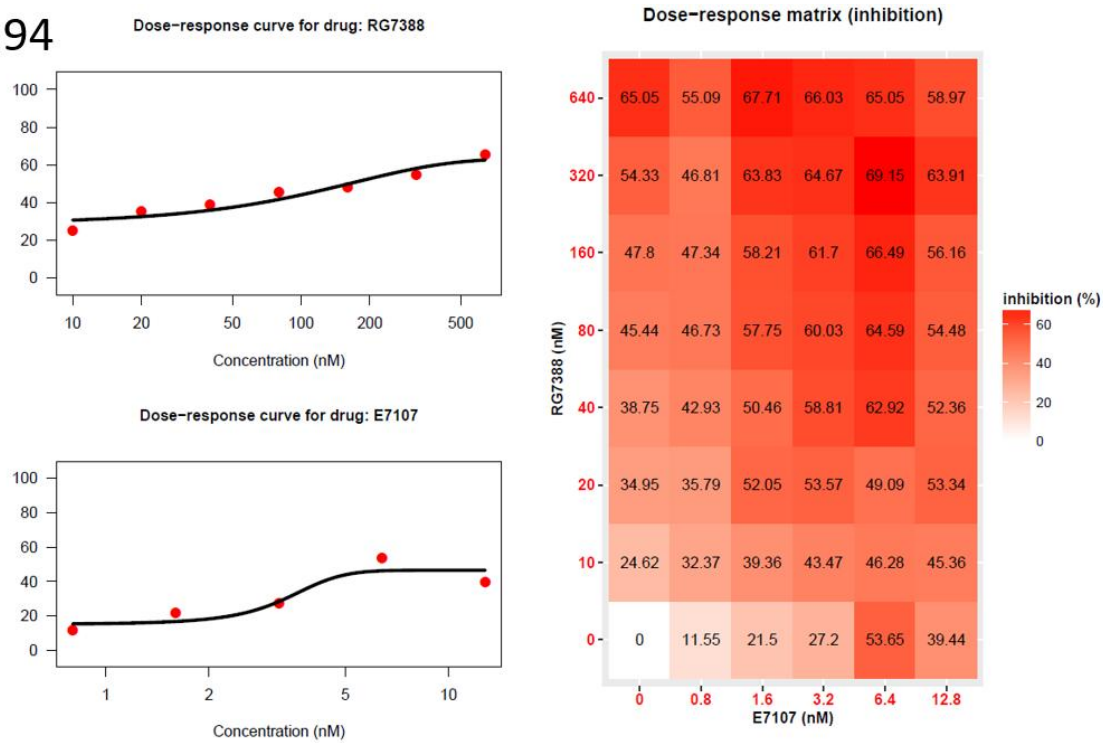

CLL305

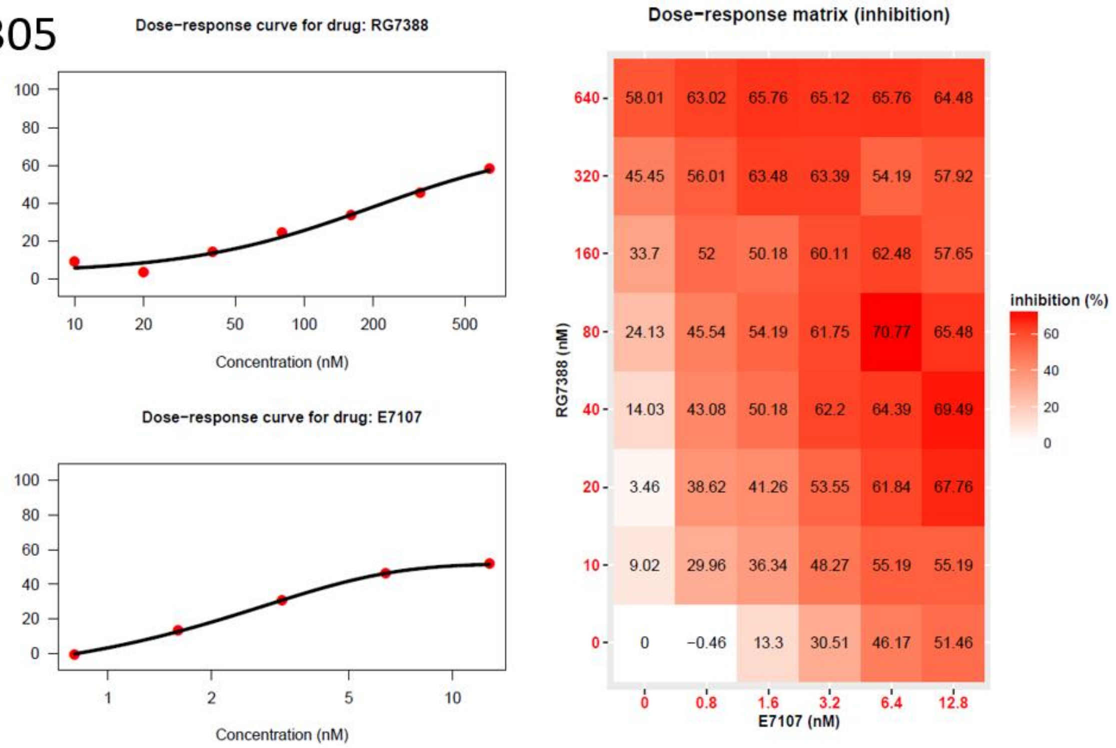

CLL301

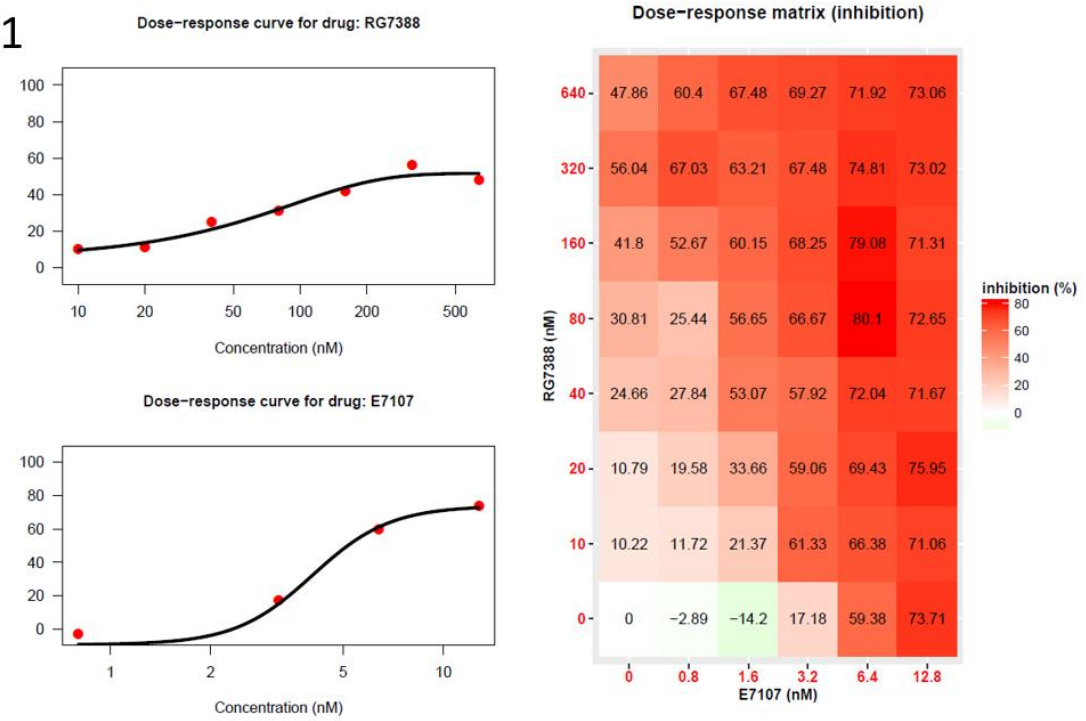

CLL308

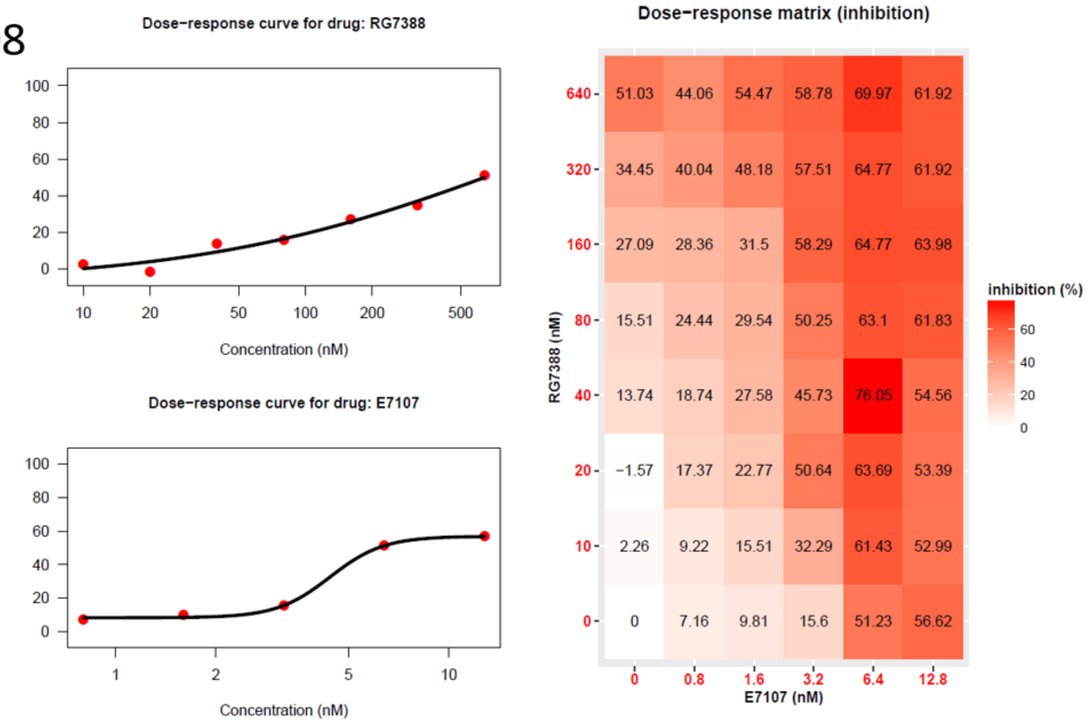

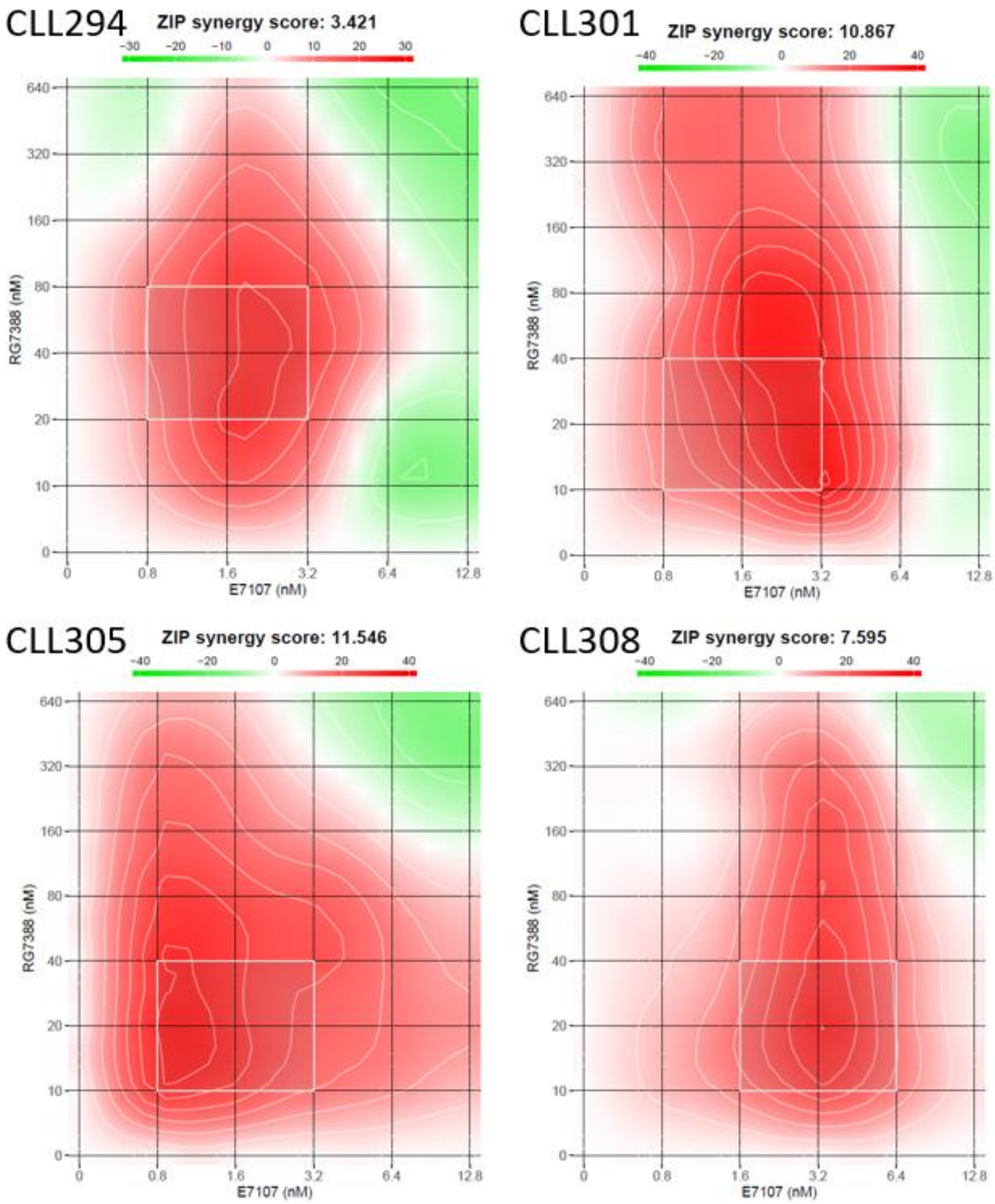
CLL294
ZIP synergy score: 3.421
CLL301
ZIP synergy score: 10.867
CLL305
ZIP synergy score: 11.546
CLL308
ZIP synergy score: 7.595
RG7388 (nM)
E7107 (nM)
0
0.8
1.6
3.2
6.4
12.8
0
10
20
40
80
160
320
640
